# A global bibliometric analysis of intimate partner violence in the field of HIV/AIDS: implications for interventions and research development

**DOI:** 10.3389/fpubh.2023.1105018

**Published:** 2023-06-15

**Authors:** Tham Thi Nguyen, Lilian Ha, Long Hoang Nguyen, Linh Gia Vu, Hoa Thi Do, Laurent Boyer, Guillaume Fond, Pascal Auquier, Carl A. Latkin, Cyrus S. H. Ho, Roger C. M. Ho

**Affiliations:** ^1^Institute for Global Health Innovations, Duy Tan University, Da Nang, Vietnam; ^2^Faculty of Medicine, Duy Tan University, Da Nang, Vietnam; ^3^Columbia University, New York, NY, United States; ^4^Department of Public Health Sciences, Karolinska Institutet, Stockholm, Sweden; ^5^Institute of Health Economics and Technology, Hanoi, Vietnam; ^6^CEReSS, Research Centre on Health Services and Quality of Life, Aix Marseille University, Marseille, France; ^7^Bloomberg School of Public Health, Johns Hopkins University, Baltimore, MD, United States; ^8^Department of Psychological Medicine, Yong Loo Lin School of Medicine, National University of Singapore, Singapore, Singapore; ^9^Institute for Health Innovation and Technology (iHealthtech), National University of Singapore, Singapore, Singapore

**Keywords:** global mapping, intimate partner violence, domestic violence, HIV/AIDS, bibliometric analysis, IPV

## Abstract

This study aimed to explore the research landscape of intimate partner violence (IPV)—harm-induced behavior in an intimate relationship and HIV/AIDS to determine lessons learnt and gaps that may be filled by future research. Publications related to IPV, and HIV/AIDS published from 1997 to 2019 were collected from Web of Science (WoS). STATA and VOSviewer software tools were used for bibliometric analysis. Content analysis, common topics, and the map of co-occurrence terms were structured by Latent Dirichlet allocation and VOSviewer software tool. 941 studies were included. Factors associated with domestic violence and interventions to reduce IPV were the two most common themes. Meanwhile, mental health illness among pregnant women affected by HIV and IPV, and HIV-risk among youth suffering from IPV have not received adequate attention. We suggest that more research focusing on adolescents and pregnant women affected by HIV and IPV. In addition, the development of collaborative networks between developed and developing countries should also be addressed.

## 1. Introduction

Intimate partner violence (IPV) is defined as a “behavior within an intimate relationship that causes physical, sexual or psychological harm, including physical aggression, sexual coercion, psychological abuse and controlling behaviors” perpetrated by a significant other (1). According to WHO, the global burden of IPV is often borne by women while the majority of perpetrators being males (1). While approximately 30% of women globally are victims of IPV (2), it may also occur against males and in same-sex relationships. The prevalence of IPV among bisexual women, lesbian, bisexual men, and homosexual men were 61.1%, 43.8%, 37.3%, and 26.0%, respectively (3).

HIV/AIDS and IPV are major public health challenges with close intersection with each other. IPV is associated with HIV transmission, as indicated by lower rates of condom use among people with IPV (4). Furthermore, male perpetrators are more likely to engage in HIV-risk sexual behaviors, such as multiple partners, condomless sex, and/or drug use (5). For people living with HIV/AIDS (PLHA), IPV was significantly associated with poor adherence to antiviral therapy (ART) (6), negative impact on the engagement of women with HIV health care services (7–11), and heightened risk of poorer mental and physical health outcomes (12, 13).

Since IPV has been linked to significant challenges for HIV care, treatment adherence, and overall quality of life for PLHA, there have been recent efforts to characterize research trends and findings on the topic of IPV and HIV/AIDS. For example, a systematic review by Kouyoumdjian et al. evaluated 101 articles, including both qualitative and quantitative studies on the relationship between IPV and HIV (14). There have also been reviews focused on characterizing IPV and HIV interventions among women in sub-Saharan Africa (15), as well as a more recent review by Marshall et al. examining the efficacy of 14 international studies on interventions that address both IPV and HIV among women (16). While the previous systematic reviews provided in detail reliable and accurate summaries, they consume time and human resources (17). Meanwhile, some authors have used bibliometrics to explore the trend of research in IPV from 2005 to 2014 (18), or gender-based violence from 1982 to 2012 (19). Both studies showed an increase in the number of papers related to IPV and indicated new research topics, such as the relationship between IPV and HIV infection among pregnant women, during the study period. However, these studies did not include publications from 2015 up to 2019, and may not fully reflect the current trend. Also, they did not use context analysis to explore research topics and research disciplines.

To the best of our knowledge, there are currently no studies that examine the existing literature on IPV in HIV/AIDS studies using a combination of topic modelling and scientometrics approach. This study aims to evaluate the productivity of research quantitatively, examine the trend and research areas, and determine a visual network of international collaboration globally as well as explore research topics and research disciplines on IPV in HIV/AIDS publications. Our results could aid researchers, health care providers, and policymakers by informing them of the direction for future studies and interventions worldwide.

## 2. Methods

### 2.1. Database and keywords

In this scientometrics study, Web of Science Core Collection (WoS) was used for data collection. It has several advantages compared with other databases, including (1) allowing for the extraction of a large number of records with fundamental information (titles, abstracts, number of citations, number of download times, and research area), and (2) coverage of references since 1900 from high impact journals worldwide. The search strategy included two steps.

First, Topic search (Title, abstract, authors’ keywords, and keywords plus) and Boolean logic AND/ OR were used for the keywords: “HIV,” “HIV and AIDS,” “Human immunodeficiency virus,” and “Acquired Immune Deficiency Syndrome.” Scientific papers from January 1, 2020 onward were excluded because the search process was conducted on March 28, 2020, and the papers up to March 2020 may not fully reflect the trend of that year. Hence, in this study, we included publications related to IPV, and HIV/AIDS published from 1997 to 2019. The publications chosen were research articles and research reviews in English. Other documents such as books, book chapters, or data papers and in any other language were excluded. The data were downloaded manually and independently by two researchers. Any inconsistencies in the results were checked by a senior researcher (e.g., missing records or missing information of data) and the downloads of the data were re-entered.

Secondly, we transferred text data into STATA for further filtering. Title and abstract search containing the phrase “Intimate partner violence” OR “domestic violence” OR “spouse violence” were extracted. Keywords for HIV and “Intimate partner violence” were contributed by health experts, Mesh terms (20), and previous studies (21). A final sample of 941 papers was used for further analysis.

### 2.2. Bibliometric analysis, text mining, and topic modelling

In this study, the bibliometric analysis was used to explore the trend in this research field. The final dataset was analyzed using STATA (version 15, StataCorp LLC, Texas, United States) by the following indicators: publication year, an annual number of publications, total and average counts of citation and download times per 6 months and 5 years. A network graph showing the collaboration among countries sharing co-authorships was visualized using VOSviewer software tool (22).

To explore the hidden topics of the dataset, a visualization tool by VOSviewer and topic modelling by Latent Dirichlet allocation (LDA), were used. In this study, the VOSviewer (version 1.6.8, Center for Science and Technology, Leiden University, Netherlands) was applied to illustrate the relationship between the most frequent terms in titles/abstracts of selected publications. Additionally, a co-occurrence network and a country network were created using this software (Table 1). The dendrogram was applied to identify the hierarchical clustering of major research disciplines from selected papers. Analytical techniques for each data type were presented in Table 1.

**Table 1:** Analytical techniques and outcomes of each data type.

Type of data	Unit of analysis	Analytical methods	Presentations of results
Keywords, countries	Words	Frequency of co-occurrence	Map of keywords clusters, countries
Abstracts	Papers	Latent Dirichlet allocation	10 classifications of research topics
WOS classification of research areas	WOS research areas	Haberman distance	Dendrogram of research disciplines

**Figure 1:**
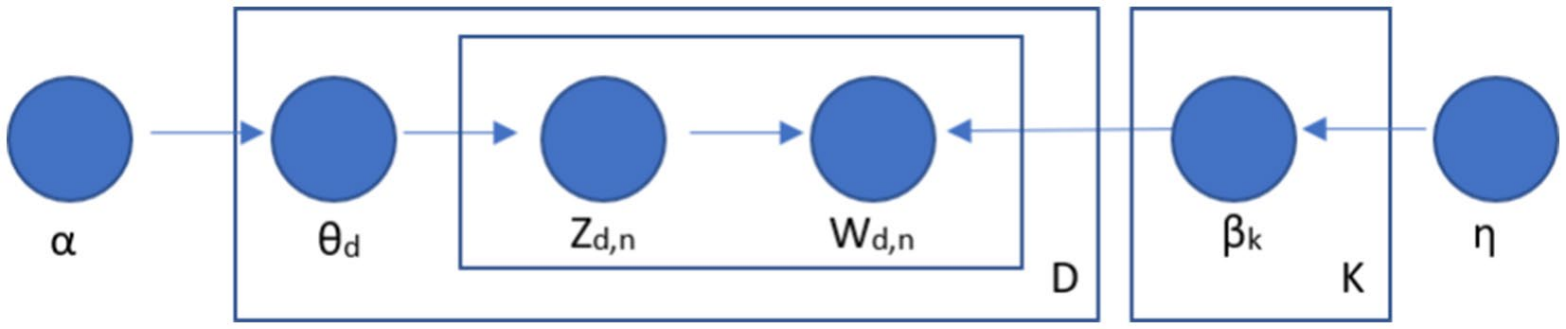
LDA’s algorithm in topic modeling. Where: *K*, number of topics; *α*, prior weight of topic k by document based on Dirichlet distributions, identifying *θ*; *η*, prior weight of word w by document based on Dirichlet distributions, identifying *β*; *θ*_d_, ratios of topics as per document; *β*_k_, probability that word w is created by topic; *Z*_d,*n*_, topic of the *n*th word in document d, created from θ_d_; *W*_d,*n*_, *n*th word in document d, determined by Z_d,*n*_.

In addiction, topic modeling was utilized by using the LDA technique to identify 10 latent topics from the titles and abstracts of selected documents (23–26). Particularly, a set of words with relevant meanings might be grouped together according to this algorithm to reflect a certain topic. LDA is a Bayesian probabilistic topic model and one of the most common techniques for topic modeling. Up until now, LDA had been defined as a state-of-art or the most common method for topic modeling (27). In particular, the LDA considers all pooled publications (e.g., Intimate partner violence and HIV/AIDS publications in this study) representing K latent topics, and each topic is also represented by a set of words (27). The LDA generative process is illustrated in Figure 1 (28).

In this study, STATA’s LDA command was applied (29). In the initial stage, STATA was used to decompose the titles and abstracts into individual words. The words are then given to one of the n themes at random with equal probability. A new subject is assigned for words with a similar theme after the burn-in time (every 50 iterations). After LDA is completed, the file in Excel format containing allocated topics was exported (30). After obtaining the outputs of the LDA model, we invited HIV and IPV experts to discuss and label the topics by providing them with words (after sorted) with the highest probability appear within each topic and titles/abstracts of papers within each topic.

## 3. Results

Table 2 reveals the characteristics of the dataset. The first paper was published in 1997. Since then, there has been a gradual increase in the number of studies on IPV among HIV/AIDS studies during 1997–2019, contributing to a total of 941 papers. Notably, the total number of download times (total usage) and the average number of downloads times (the mean use rate) in the last 5 years shows the middle-term interest of readers. These highest figures belonged to that of papers published in 2013. Also, total usage and mean use rate in the last 6 months were highest for papers published in 2019, which shows the recent increased interest by readers.

**Table 2:** General characteristics of publications (total 941).

Year published	Total number of papers	Total citations	Mean cite rate per year	Total usage last 6 month	Total usage last 5 years	Mean use rate last 6 month	Mean use rate last 5 year
2019	105	100	1.0	182	372	1.7	0.7
2018	103	352	1.7	63	552	0.6	1.1
2017	91	570	2.1	34	714	0.4	1.6
2016	107	1,072	2.5	41	1,213	0.4	2.3
2015	87	1,786	4.1	57	1,619	0.7	3.7
2014	77	1,710	3.7	33	1,397	0.4	3.6
2013	75	1,732	3.3	29	1,555	0.4	4.1
2012	41	1,440	4.4	17	724	0.4	3.5
2011	50	1,881	4.2	18	750	0.4	3.0
2010	40	2,095	5.2	9	600	0.2	3.0
2009	33	1,217	3.4	12	419	0.4	2.5
2008	33	2,251	5.7	11	459	0.3	2.8
2007	19	1,349	5.5	9	332	0.5	3.5
2006	18	1,673	6.6	3	257	0.2	2.9
2005	16	736	3.1	3	99	0.2	1.2
2004	11	1,185	6.7	16	247	1.5	4.5
2003	10	908	5.3	4	106	0.4	2.1
2002	5	272	3.0	0	34	0.0	1.4
2001	4	222	2.9	0	28	0.0	1.4
2000	8	725	4.5	3	72	0.4	1.8
1999	5	73	0.7	0	16	0.0	0.6
1998	2	205	4.7	0	21	0.0	2.1
1997	1	156	6.8	0	33	0.0	6.6

**Figure 2:**
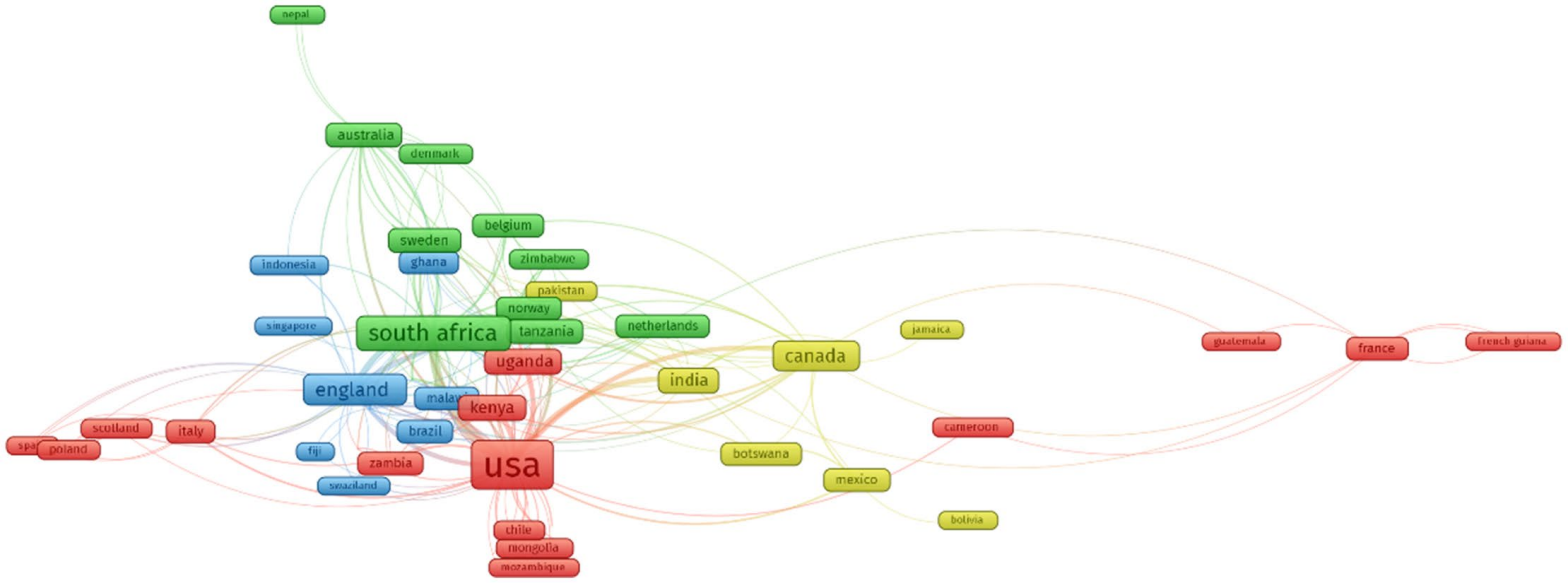
Collaboration of countries regarding intimate partner violence among people living with HIV during 1997–2019 (Red cluster: the US and sub-Saharan African countries; green cluster: South Africa and North European countries; blue cluster: England, Brazil, and Southeast Asia countries).

Figure 2 shows the collaboration of countries in HIV/AIDS research mentioning intimate partner violence. There were 65 countries with at least one paper in the figure. There were four main groups in this research field, including (1) the U.S and sub-Saharan African countries (red cluster), (2) South Africa and North European countries (green cluster), and (3) England, Brazil, and Southeast Asia countries (blue cluster).

**Figure 3:**
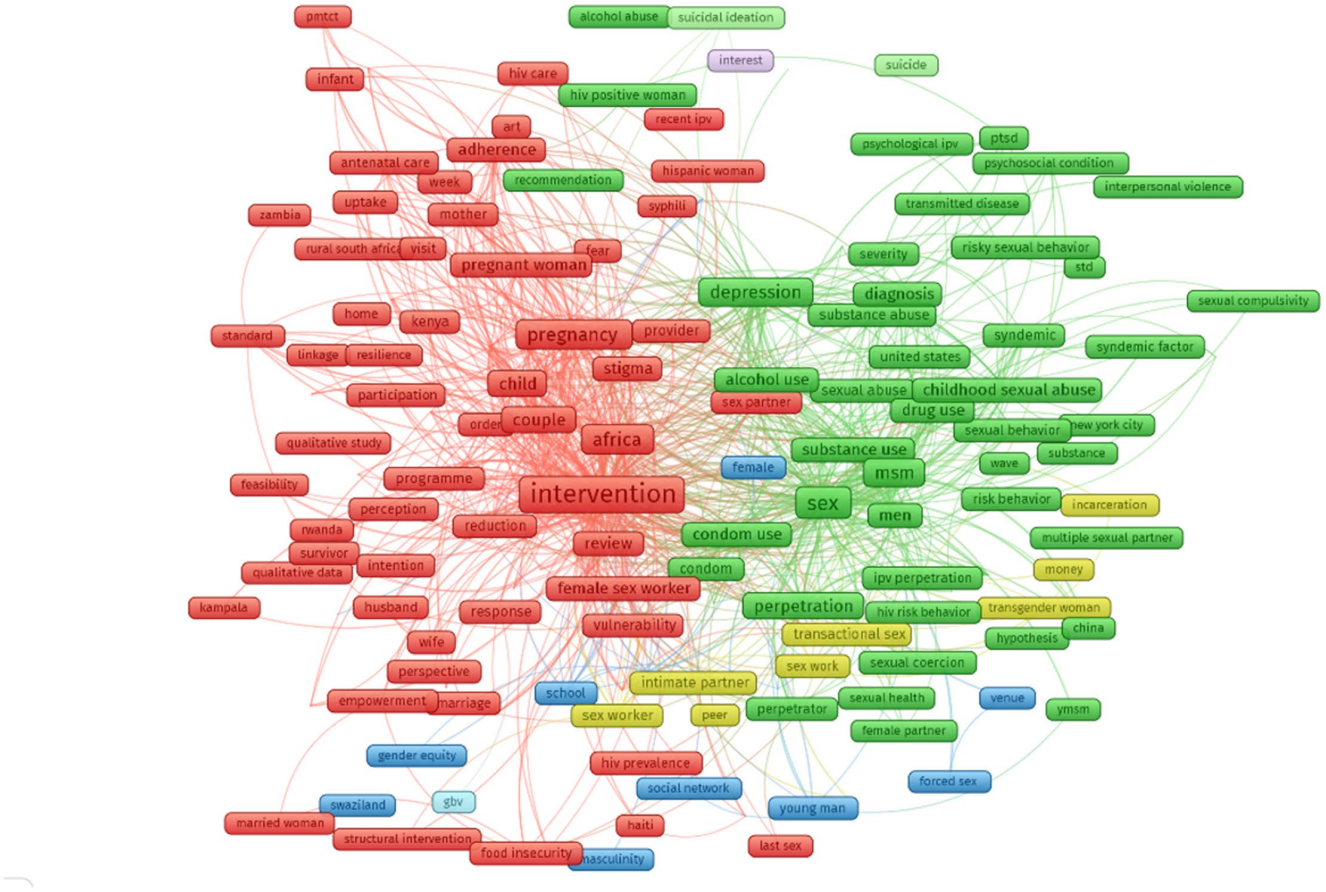
Co-occurrence of most frequent terms in titles and abstract (The size of the term was calculated based on its weight. The color was determined by its cluster. The length of lines showed the relatedness between two terms; red cluster: interventions to reduce intimate partner violence; green cluster: factors associated with IPV; yellow cluster: IPV and sex workers; blue cluster: gender inequity in IPV).

By using the text mining function of VOSwiver, the co-occurrence of terms was visualized, with the most frequent groups of terms displayed in Figure 3. There were two major clusters, (1) Cluster 1 (red) refers to interventions to reduce intimate partner violence and (2) Cluster 2 (green) focuses on factors associated with IPV. The two minor clusters included IPV and sex workers (yellow cluster), and gender inequity in IPV (blue cluster).

Table 3 shows the most cited papers with more than 200 citations among all papers in the analysis. The titles and abstracts of the publications were carefully reviewed and assigned to a suitable topic based on LDA. Among those 10 papers, gender inequity, and factors associated with domestic violence were the most common topics. The two most cited papers in our dataset addressed the high risk of HIV infection among women suffering from violence from male partners (31), especially among young South African women (32). The third paper in our list evaluated the risk factors affecting IPV against women (33).

**Table 3:** Most cited papers.

No	Topic	Authors	Title	Journal	Total cite	Publication year	Cite rate per year
1	Topic 5	Dunkle KL et al.	Gender-based violence, relationship power, and risk of HIV infection in women attending antenatal clinics in South Africa	Lancet	781	2004	48.8
2	Topic 5	Jewkes RK et al.	Intimate partner violence, relationship power inequity, and incidence of HIV infection in young women in South Africa: a cohort study	Lancet	627	2010	62.7
3	Topic 2	Abramsky T et al.	What factors are associated with recent intimate partner violence? findings from the WHO multi-country study on women’s health and domestic violence	BMC Public Health	428	2011	47.6
4	Topic 2	Jewkes R et al.	Impact of steppingstone on incidence of HIV and HSV-2 and sexual behavior in rural South Africa: cluster randomized controlled trial	BMJ-British Medical Journal	426	2008	35.5
5	Topic 5	Jewkes R et al.	Gender and sexuality: emerging perspectives from the heterosexual epidemic in South Africa and implications for HIV risk and prevention	Journal of The International Aids Society	334	2010	33.4
6	Topic 2	Kim JC et al.	Understanding the impact of a microfinance-based intervention on women’s empowerment and the reduction of intimate partner violence in South Africa	American Journal of Public Health	268	2007	20.6
7	Topic 5	Jewkes RK et al.	Gender inequalities, intimate partner violence and HIV preventive practices: findings of a South African cross-sectional study	Social Science & Medicine	241	2003	14.2
8	Topic 6	Cohen M et al.	Domestic violence and childhood sexual abuse in HIV-infected women and women at risk for HIV	American Journal of Public Health	238	2000	11.9
9	Topic 2	Coker AL	Does physical intimate partner violence affect sexual health? A systematic review	Trauma Violence & Abuse	228	2007	17.5
10	Topic 7	Dunkle KL et al.	Perpetration of partner violence and HIV risk behavior among young men in the rural Eastern Cape, South Africa	AIDS	207	2006	14.8

Topic 2: prevalence and factors associated with domestic violence; Topic 5: health services and women suffered intimate partner violence; Topic 6: mental health illness and domestic violence among pregnant women infected with HIV/AIDS; Topic 7: prevalence and factors associated with domestic violence.

Latent Dirichlet allocation classified titles and abstracts of the dataset into 10 research topics (Table 4). Each topic was identified upon careful review of the titles and abstracts as well as the most frequently appearing words. After that, based on experts’ opinions, it was decided to merge topic 2, topic 4, and topic 7 into one new topic (topic 2), and topic 1 and topic 3 into topic 1, as they share the same theme. Factors associated with domestic violence among people living with HIV had the highest number of papers during 1997–2019 and in the last 5 years, with 272 papers and 145 papers, respectively. Additionally, interventions to reduce domestic violence and health care services for women with domestic violence also received high concern from scientists. Youth and pregnant women suffered a large impact on domestic violence, particularly in families affected by HIV/AIDS (34). The number of papers concerning these two subjects in the last 5 years contributed 65.2% and 74.6%, respectively, showing an increased research interest in these topics.

**Table 4:** Ten research topics classified by LDA.

Topic	Research areas	Frequency	Percent (of total 832 papers)	Frequency (2014–2019)	Percent (% total of each topic)
Topic 1	Interventions and preventions domestic violence	79	9.5	50	61.6
Topic 2	Prevalence and Factors associated with domestic violence	78	9.4	57	53.3
Topic 3	Interventions and preventions domestic violence	85	10.2	51	–
Topic 4	Prevalence and Factors associated with domestic violence	6	0.7	3	–
Topic 5	Health services and women suffered intimate partner violence	70	8.4	32	45.7
Topic 6	Mental health illness and domestic violence among pregnant women infected with HIV/AIDS	59	7.1	44	74.6
Topic 7	Prevalence and Factors associated with domestic violence	188	22.6	85	–
Topic 8	Relationship power inequity, social norms, and intimate domestic partner	102	12.3	55	53.9
Topic 9	Youth, HIV risks and domestic violence	23	2.8	15	65.2
Topic 10	Perpetration of intimate partner violence and sexual risk behaviors	142	17.1	50	35.3
		**832**	**100**	**442**	

**Figure 4:**
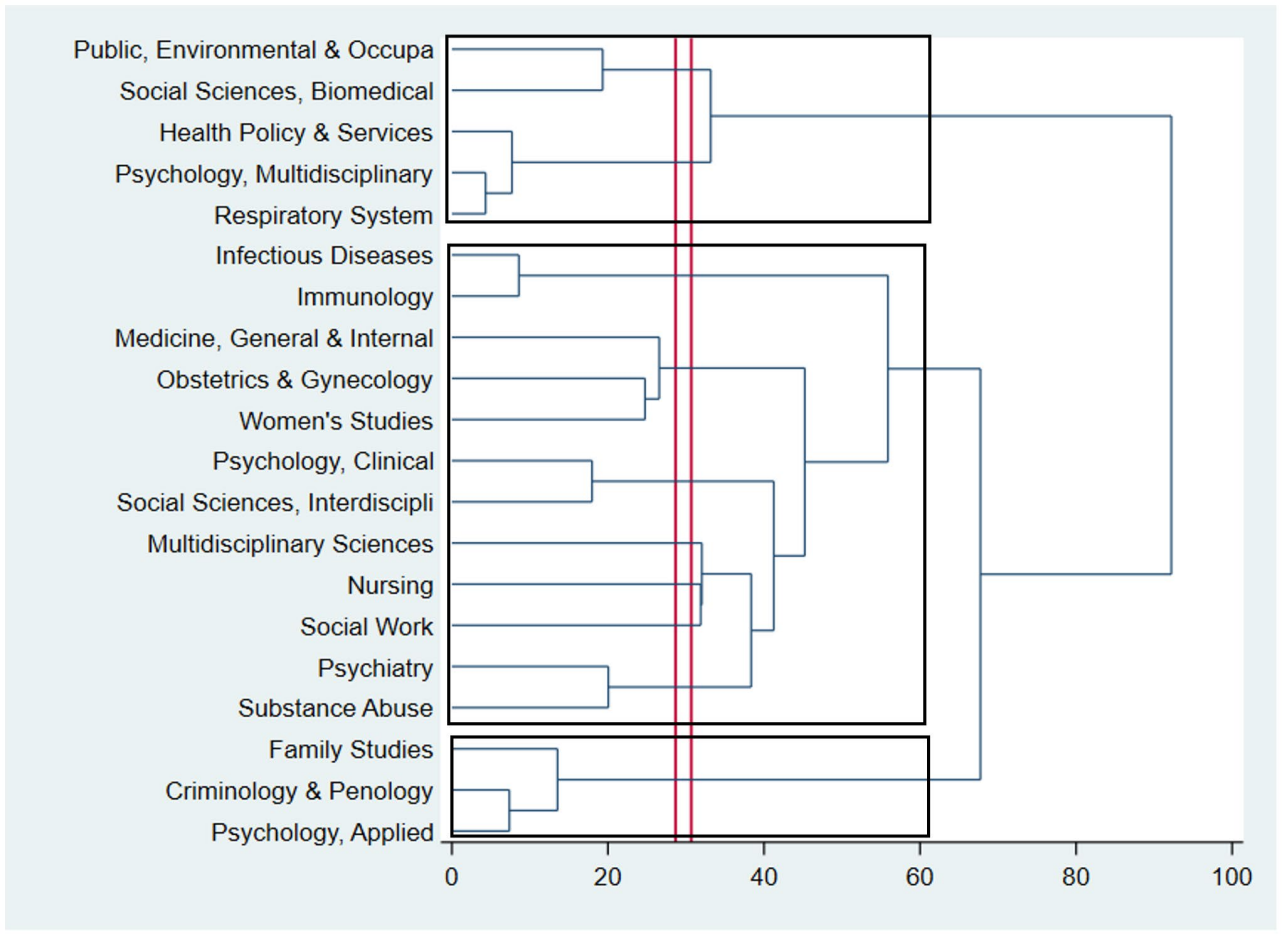
Dendrogram of coincidence of research areas using the WoS classifications.

Figure 4 shows the hierarchical clustering of research areas in IPV in HIV/AIDS research. The dissimilarity between research areas was shown by the horizontal axis; meanwhile, research areas were presented by the vertical axis. The red lines show the depth of the cut-off for each cluster. There were three main categories of research areas regarding domestic violence among HIV/AIDS publications. They were (1) social sciences and environment-related health issues, (2) criminological psychology and family, and the third with six sub-clusters: (a) Psychology, respiratory healthcare, and health policy, (b) Immunology of Infectious Diseases, (c) Obstetrics, Gynecology, and Women’s Health, (d) Social Sciences, and Psychology, (e) Healthcare, and (f) Mental health and substance misuse. As can be seen, the research areas of Psychology, Psychiatry, Social Sciences, and Women’s Health garnered great concern among researchers. However, there was limited research in areas focusing on children or health care services for those suffering from domestic violence, as well as on economic aspects of these studies.

## 4. Discussion

This study provides an overview of the trend in published works as well as common topics regarding intimate violence studies in HIV/AIDS publications from 1997 to 2019. The findings showed that most papers are published and conducted in developed countries, led by the US. Two most common research themes were factors associated with domestic violence and intervention and prevention to reduce domestic violence. This study emphasized that more research collaboration should be conducted between developed and developing countries. Also, more research and attention should be paid to mental health issues and domestic violence among pregnant women infected with HIV/AIDS, as well as the relationship between HIV risks and domestic violence among youth.

In accordance with the results of other studies, research on domestic violence in HIV/AIDS publications has increased gradually during the 1997–2019 period (35, 36). In addition, most published works were contributed by developed countries and led by the U.S, in line with results from previous studies (37, 38). Up until now, Intimate partner violence (IPV) and HIV/AIDS are complex issues that affect individuals and communities worldwide, but they may manifest differently in developed and developing countries. Particularly, it should be noted that there has been a great burden of HIV/AIDS and IPV in developing countries, especially in sub-Saharan Africa (39). The prevalence of IPV among PLHA in developing countries was shown to be particularly high compared with HIV-negative populations (14, 40–44). For example, the prevalence of violence among women with HIV in Nepal was 93% (45). This number was much higher than that of Uganda (44.2%) (41), Nigeria (23.6%) (42), or the US (35%) (43). This finding suggests that the contribution of developing countries in research did not meet the need to address IPV. This dearth is likely due to the lack of resources, funding, or facilities in poor-resource countries (46). Although this gap cannot be filled in a short time, poor-resource settings should be active in research collaboration with developed countries, as well as increase internal funding and spending for HIV/AIDS research (47).

From the results of the term co-occurrence map and the research topic tables, our study affirms previous reviews which show various approaches related to HIV/AIDS and IPV research, including prevalence and associated factors, gender inequity, and domestic violence (48), and studies focused on interventions or prevention of intimate partner violence (15). With the high burden of disease due to the intersection of HIV and IPV, it is no doubt that research addressing the prevention of IPV, as well as evaluation of the effectiveness of interventions, are needed. In our dataset, most studies focused on some intervention that addresses IPV and women affected with HIV/AIDS, such as the Women’s Health CoOp (WHC) (49), SASA! Activist kit (50), or microfinance intervention (51). A similar conclusion was reached by Marshall KJ et al. (16). The above interventions addressed HIV and IPV with different approaches, target populations, and outcomes. For example, a group that received microfinance and training intervention showed better economic well-being, reducing HIV risk behavior compared with the control group (51). Similarly, Wechsberg et al. showed that the Women’s Health CoOp brief peer-facilitated intervention was effective in promoting drug abstinence among women using drugs in South Africa.

In addition, the results show evidence of the limited number of papers mentioning mental health illness and domestic violence among pregnant women infected with HIV/AIDS, compared to other topics. However, 74.6% of published works mentioning this vulnerable population were conducted in the last 5 years. This phenomenon indicates that the concern of scientists and policymakers has been transferred from HIV high-risk populations, such as sex workers, to this vulnerable population (52). This could be partially explained by the serious health problems which IPV poses to pregnant women affected by HIV, such as mental health issues, poor child health outcomes (53), or depression and suicidal ideation. A study from Kapetanovic S, et al. also confirmed this result (53). Moreover, 65% (15/23 studies) of studies concerning adolescents, HIV infection, and domestic violence were conducted in the last 5 years. This result highlights that there has been a growing recognition that adolescents are vulnerable to HIV risks and domestic violence (54). Most of the studies regarding these two vulnerable populations were conducted in sub-Saharan Africa countries, where women and youth face higher risks of HIV infection when they have suffered violence, compared with those who have not (31, 55). Furthermore, the thematic map showed that there was a limitation in the number of studies mentioning violence among sex workers, although the relationship between IPV and HIV risk among sex workers has been well-documented in previous studies (56, 57).

Some implications can be derived from this study. First, future research may benefit from viewing IPV and HIV/AIDS as an intersecting healthcare challenges when assessing related factors and interventions for these two problems (58). For example, future research may focus on the cost-analysis of interventions targeting IPV and HIV-related outcomes (59). Secondly, support and research collaborations between developed countries and developing countries could bring more fruitful results to increase the health status of PLWHA and IPV, especially in regions with a high prevalence of HIV. Moreover, due to the growing number of PLWHA and the association between HIV and IPV, comprehensive research should be conducted not only on prevalence, associated factors and interventions or prevention, but also focused on healthcare service, which is a priority in public health, especially among pregnant women and adolescents with HIV who suffer IPV (60). Although women and girls experience a higher risk for IPV, many boys also suffer from physical, sexual, and emotional domestic violence (61). Future studies thus may consider taking a focus on male victims of IPV. In addition, further research is required to access for post-traumatic stress disorder as well as mental health conditions among the victims.

Considering these findings, several limitations of the study should be considered. Firstly, the shortcoming of this study was that only one database from the Web of Sciences was used. Despite a number of journals in the field of HIV indexed in the Web of Science database, we acknowledge that it is still not fully representative of all databases. And this might restrict our ability to cover all relevant publications as well as certain disciplines may not be included in this study. Secondly, only English-language publications were selected in this study, which may not fully reflect the trend of the research field because in some developing countries, publications may not have been not written in English. Also, only titles and abstracts were used for text mining. However, different levels of data, including countries, text data visualization, topic modelling, and research disciplines, were used to increase the results of this research. Finally, 10 topics from the LDA model were labeled based on HIV and IPV experts. This may exist some biases as well as can be affected the subjectivity of the results.

## 5. Conclusion

Our study highlighted a gradual increase of publications regarding IPV research in HIV/AIDS studies during the 1997–2019 period. Associated factors of IPV and prevention and intervention to reduce IPV among HIV/AIDS were the most common topics. Meanwhile, there was a limitation in the number of published works related to mental health illness, pregnant women, adolescents, and sex workers who suffer from IPV. More future studies should focus on these l topics with the interdisciplinary collaboration of research fields. In addition, support and research collaboration between developed countries and developing countries might be a key element to reduce the global burden of this overlapping health issue.

## Author contributions

TN, LH, LN, and CL: conceptualization. LN and RH: methodology. TN, LV, HD, and CH: formal analysis and investigation. LH, LN, TN, LV, HD, LB, GF, PA, CL, CH, and RH: writing—original draft preparation and writing—review and editing. LB, GF, PA, CL, CH, RH, LH, and HD: supervision. All authors contributed to the article and approved the submitted version.

## Funding

This study was funded by NUS iHeathtech Other Operating Expenses (R-722-000-004-731) and NUS Department of Psychological Medicine (R-177-000-100-001/R-177-000-003-001).

## Conflict of interest

The authors declare that the research was conducted in the absence of any commercial or financial relationships that could be construed as a potential conflict of interest.

## Publisher’s note

All claims expressed in this article are solely those of the authors and do not necessarily represent those of their affiliated organizations, or those of the publisher, the editors and the reviewers. Any product that may be evaluated in this article, or claim that may be made by its manufacturer, is not guaranteed or endorsed by the publisher.

## References

[1] WHO. Preventing intimate partner and sexual violence against women: Taking action and generating evidence. Geneva: World Health Organization (2010). 10.1136/ip.2010.029629 20921563

[2] García-Moreno C Pallitto C Devries K Stöckl H Watts C Abrahams N. Global and regional estimates of violence against women: prevalence and health effects of intimate partner violence and non-partner sexual violence. Geneva: World Health Organization (2013).

[3] Walters ML Chen J Breiding MJ. The National Intimate Partner and sexual violence survey (NISVS): 2010 findings on victimization by sexual orientation. Atlanta, GA: National Center for Injury Prevention and Control, Centers for Disease Control and Prevention (2013).

[4] Decker MR Miller E McCauley HL Tancredi DJ Anderson H Levenson RR. Recent partner violence and sexual and drug-related STI/HIV risk among adolescent and young adult women attending family planning clinics. Sex Transm Infect. (2014) 90:145–9. doi: 10.1136/sextrans-2013-051288 24234072 PMC4305329

[5] Mumtaz GR Weiss HA Thomas SL Riome S Setayesh H Riedner G. HIV among people who inject drugs in the Middle East and North Africa: systematic review and data synthesis. PLoS Med. (2014) 11:e1001663. doi: 10.1371/journal.pmed.1001663 24937136 PMC4061009

[6] Hatcher AM Smout EM Turan JM Christofides N Stöckl H. Intimate partner violence and engagement in HIV care and treatment among women. AIDS. (2015) 29:2183–94. doi: 10.1097/QAD.0000000000000842 26353027

[7] Bryan N Davidov DM Dick T Bassler J Fisher M. Intimate partner violence experiences among men living with HIV in rural Appalachia. AIDS Behav. (2019) 23:3002–14. doi: 10.1007/s10461-019-02438-3 30924062 PMC6765448

[8] Hatcher A Smout E Turan J Christofides N Stöckl H. Intimate partner violence and engagement in HIV care and treatment among women: a systematic review and meta-analysis. AIDS. (2015) 29:2183–94. doi: 10.1097/QAD.0000000000000842 26353027

[9] Leddy A Weiss E Yam E Pulerwitz J. Gender-based violence and engagement in biomedical HIV prevention, care and treatment: a scoping review. BMC Public Health. (2019) 19:897. doi: 10.1186/s12889-019-7192-4 31286914 PMC6615289

[10] Siemieniuk R Krentz H Gill M. Intimate partner violence and HIV: a review. Curr HIV/AIDS Rep. (2013) 10:380–9. doi: 10.1007/s11904-013-0173-9 23943348

[11] Sullivan T. The intersection of intimate partner violence and HIV: detection, disclosure, discussion, and implications for treatment adherence. Top Antivir Med. (2019) 27:84. 31136996 PMC6550354

[12] Ellsberg M Jansen H Heise L Watts C Garcia-Moreno C. Intimate partner violence and women's physical and mental health in the WHO multi-country study on women's health and domestic violence: an observational study. Lancet (London, England). (2008) 371:1165–72. doi: 10.1016/S0140-6736(08)60522-X 18395577

[13] Lövestad S Löve J Vaez M Krantz G. Prevalence of intimate partner violence and its association with symptoms of depression; a cross-sectional study based on a female population sample in Sweden. BMC Public Health. (2017) 17:335. doi: 10.1186/s12889-017-4222-y 28424072 PMC5397670

[14] Kouyoumdjian F Findlay N Schwandt M Calzavara L. A systematic review of the relationships between intimate partner violence and HIV/AIDS. PLoS One [Internet]. (2013) 8:e81044. doi: 10.1371/journal.pone.0081044 24282566 PMC3840028

[15] Anderson J Campbell J Farley J. Interventions to address HIV and intimate partner violence in sub-Saharan Africa: a review of the literature. J Assoc Nurses AIDS Care. (2013) 24:383–90. doi: 10.1016/j.jana.2013.03.003 23790280 PMC3694280

[16] Marshall K Fowler D Walters M Doreson A. Interventions that address intimate partner violence and HIV among women: a systematic review. AIDS Behav. (2018) 22:3244–63. doi: 10.1007/s10461-017-2020-2 29313192 PMC6035885

[17] University of California San Francisco. Systematic Review 2020 Available at: https://guides.ucsf.edu/c.php?g=375744&p=3151000

[18] Wang H Xu J Shang H Wang N. Bibliometric analysis of worldwide literature in research of intimate partner violence in the world in recent 10 years. Zhonghua Liu Xing Bing Xue Za Zhi. (2015) 36:1172–5. 26837368

[19] Brilhante AVM Moreira GAR Vieira LJES Catrib AMF. Um estudo bibliométrico sobre a violência de gênero. Saúde Soc. (2016) 25:703–15. doi: 10.1590/s0104-12902016148937

[20] Medical Subject Headings M. Spouse Abuse. Available at: https://www.ncbi.nlm.nih.gov/mesh/68013179.

[21] Trabold N McMahon J Alsobrooks S Whitney S Mittal M. A systematic review of intimate partner violence interventions: state of the field and implications for practitioners. Trauma Violence Abuse. (2020) 21:311–25. doi: 10.1177/1524838018767934 29649966

[22] Van Eck N Waltman L. Software survey: VOSviewer, a computer program for bibliometric mapping. Scientometrics. (2010) 84:523–38. doi: 10.1007/s11192-009-0146-3 20585380 PMC2883932

[23] Chen C Zare A Trinh HN Omotara GO Cobb JT Lagaunne TA. Partial membership latent Dirichlet allocation for soft image segmentation. IEEE Trans Image Process. (2017) 26:5590–602. doi: 10.1109/TIP.2017.2736419 28792897

[24] Lu HM Wei CP Hsiao FY. Modeling healthcare data using multiple-channel latent Dirichlet allocation. J Biomed Inform. (2016) 60:210–23. doi: 10.1016/j.jbi.2016.02.003 26898516

[25] Gross A Murthy D. Modeling virtual organizations with latent Dirichlet allocation: a case for natural language processing. Neural Netw. (2014) 58:38–49. doi: 10.1016/j.neunet.2014.05.008 24930023

[26] Blei DM Ng AY Jordan MI. Latent dirichlet allocation. J Mach Learn Res. (2003) 3:993–1022.

[27] Asmussen CB Møller C. Smart literature review: a practical topic modelling approach to exploratory literature review. J Big Data. (2019) 6:93. doi: 10.1186/s40537-019-0255-7

[28] Kang HJ Kim C Kang K. Analysis of the trends in biochemical research using latent Dirichlet allocation (LDA). Processes. (2019) 7:379. doi: 10.3390/pr7060379

[29] Schwarz C. Ldagibbs: a command for topic modeling in Stata using latent Dirichlet allocation. Stata J. (2018) 18:101–17. doi: 10.1177/1536867X1801800107

[30] Blei DM Ng AY Jordan MI. “Latent dirichlet allocation” in Proceedings of the 14th International Conference on Neural Information Processing Systems: Natural and Synthetic. Vancouver, British Columbia, Canada: MIT Press. (2001) 601–8

[31] Dunkle KL Jewkes RK Brown HC Gray GE McIntryre JA Harlow SD. Gender-based violence, relationship power, and risk of HIV infection in women attending antenatal clinics in South Africa. Lancet. (2004) 363:1415–21. doi: 10.1016/S0140-6736(04)16098-4 15121402

[32] Jewkes RK Dunkle K Nduna M Shai N. Intimate partner violence, relationship power inequity, and incidence of HIV infection in young women in South Africa: a cohort study. Lancet. (2010) 376:41–8. doi: 10.1016/S0140-6736(10)60548-X 20557928

[33] Abramsky T Watts C Garcia-Moreno C Devries K Kiss L Ellsberg M. What factors are associated with recent intimate partner violence? Findings from the WHO multi-country study on Women's health and domestic violence. BMC Public Health. (2011) 11:109. doi: 10.1186/1471-2458-11-109 21324186 PMC3049145

[34] Skeen S Macedo A Tomlinson M Hensels I Sherr L. Exposure to violence and psychological well-being over time in children affected by HIV/AIDS in South Africa and Malawi. AIDS Care. (2016) 28:16–25. doi: 10.1080/09540121.2016.1146219 27002770 PMC4828604

[35] Bogart LM Collins RL Cunningham W Beckman R Golinelli D Eisenman D. The association of partner abuse with risky sexual behaviors among women and men with HIV/AIDS. AIDS Behav. (2005) 9:325–33. doi: 10.1007/s10461-005-9006-1 16091853

[36] Burke JG Thieman LK Gielen AC O’Campo P McDonnell KA. Intimate partner violence, substance use, and HIV among low-income women: taking a closer look. Violence Against Women. (2005) 11:1140–61. doi: 10.1177/1077801205276943 16049104

[37] Tran BX Hoang CL Tam W Phan HT Vu GT Latkin C. A global bibliometric analysis of antiretroviral treatment adherence: implications for interventions and research development (GAPRESEARCH). AIDS Care. (2020) 32:637–44. doi: 10.1080/09540121.2019.1679708 31640392

[38] Tran BX Nguyen LH Turner HC Nghiem S Vu GT Nguyen CT. Economic evaluation studies in the field of HIV/AIDS: bibliometric analysis on research development and scopes (GAP RESEARCH). BMC Health Serv Res. (2019) 19:834. doi: 10.1186/s12913-019-4613-0 31727059 PMC6854742

[39] Dwyer-Lindgren L Cork MA Sligar A Steuben KM Wilson KF Provost NR. Mapping HIV prevalence in sub-Saharan Africa between 2000 and 2017. Nature. (2019) 570:189–93. doi: 10.1038/s41586-019-1200-9, PMID: 31092927 PMC6601349

[40] Were E Curran K Delany-Moretlwe S Nakku-Joloba E Mugo NR Kiarie J. A prospective study of frequency and correlates of intimate partner violence among African heterosexual HIV serodiscordant couples. AIDS (London, England). (2011) 25:2009–18. doi: 10.1097/QAD.0b013e32834b005d, PMID: 21811146 PMC3718250

[41] Kabwama SN Bukenya J Matovu JKB Gwokyalya V Makumbi F Beyeza-Kashesya J. Intimate partner violence among HIV positive women in care – results from a national survey, Uganda 2016. BMC Womens Health. (2019) 19:130. doi: 10.1186/s12905-019-0831-1, PMID: 31675977 PMC6823960

[42] Olowookere SA Fawole OI Adekanle DA Adeleke NA Abioye-Kuteyi EA. Patterns and correlates of intimate partner violence to women living with HIV/AIDS in Osogbo. Southwest Nigeria Violence Against Women. (2015) 21:1330–40. doi: 10.1177/1077801215594889, PMID: 26175518

[43] Zierler S Witbeck B Mayer K. Sexual violence against women living with or at risk for HIV infection. Am J Prev Med. (1996) 12:304–10. doi: 10.1016/S0749-3797(18)30283-6 8909637

[44] Andrews C Reuter TK Marsh L Velazquez JM Jaoko W Jolly P. Intimate partner violence, human rights violations, and HIV among women in Nairobi. Kenya Health Hum Rights. (2020) 22:155–66. PMID: 33390704 PMC7762921

[45] Aryal N Regmi PR Mudwari NR. Violence against women living with HIV: a cross sectional study in Nepal. Global J Health Sci. (2012) 4:117. doi: 10.5539/gjhs.v4n3p117 PMC4776914 22980238

[46] Nambiar B Hargreaves DS Morroni C Heys M Crowe S Pagel C. Improving health-care quality in resource-poor settings. Bull World Health Organ. (2017) 95:76–8. doi: 10.2471/BLT.16.170803, PMID: 28053367 PMC5180347

[47] Dieleman JL Haakenstad A Micah A Moses M Abbafati C Acharya P. Spending on health and HIV/AIDS: domestic health spending and development assistance in 188 countries, 1995–2015. Lancet. (2018) 391:1799–829. doi: 10.1016/S0140-6736(18)30698-6, PMID: 29678342 PMC5946845

[48] Campbell JC Baty ML Ghandour RM Stockman JK Francisco L Wagman J. The intersection of intimate partner violence against women and HIV/AIDS: a review. Int J Inj Control Saf Promot. (2008) 15:221–31. doi: 10.1080/17457300802423224, PMID: 19051085 PMC3274697

[49] Wechsberg WM Jewkes R Novak SP Kline T Myers B Browne FA. A brief intervention for drug use, sexual risk behaviours and violence prevention with vulnerable women in South Africa: a randomised trial of the Women’s health CoOp. BMJ Open. (2013) 3:e002622. doi: 10.1136/bmjopen-2013-002622, PMID: 23793683 PMC3657672

[50] Abramsky T Devries KM Michau L Nakuti J Musuya T Kyegombe N. The impact of SASA!, a community mobilisation intervention, on women's experiences of intimate partner violence: secondary findings from a cluster randomised trial in Kampala. Uganda J Epidemiol Community Health. (2016) 70:818–25. doi: 10.1136/jech-2015-206665, PMID: 26873948 PMC4975800

[51] Pronyk PM Kim JC Abramsky T Phetla G Hargreaves JR Morison LA. A combined microfinance and training intervention can reduce HIV risk behaviour in young female participants. AIDS. (2008) 22:1659–65. doi: 10.1097/QAD.0b013e328307a040, PMID: 18670227

[52] Strathdee SA Philbin MM Semple SJ Pu M Orozovich P Martinez G. Correlates of injection drug use among female sex workers in two Mexico-U.S. border cities. Drug Alcohol Depend. (2008) 92:132–40. doi: 10.1016/j.drugalcdep.2007.07.001, PMID: 17714888 PMC2213538

[53] Kapetanovic S Dass-Brailsford P Nora D Talisman N. Mental health of HIV-seropositive women during pregnancy and postpartum period: a comprehensive literature review. AIDS Behav. (2014) 18:1152–73. doi: 10.1007/s10461-014-0728-9, PMID: 24584458 PMC4120872

[54] WHO. Violence against women and HIV/AIDS: critical intersections. Intimate partner violence and HIV/AIDS. Information Bulletin Series. (2004) 1:1–9.

[55] Maman S Mbwambo JK Hogan NM Kilonzo GP Campbell JC Weiss E. HIV-positive women report more lifetime partner violence: findings from a voluntary counseling and testing clinic in Dar Es Salaam. Tanzania Am J Public Health. (2002) 92:1331–7. doi: 10.2105/AJPH.92.8.1331, PMID: 12144993 PMC1447239

[56] Wechsberg WM Luseno WK Lam WK. Violence against substance-abusing South African sex workers: intersection with culture and HIV risk. AIDS Care. (2005) 17:55–64. doi: 10.1080/09540120500120419 16096118

[57] Ulibarri MD Strathdee SA Lozada R Magis-Rodriguez C Amaro H O'Campo P. Intimate partner violence among female sex Workers in two Mexico-U.S. border cities: partner characteristics and HIV risk-behaviors as correlates of abuse. Psychol Trauma Theory Res Pract Policy. (2010) 2:318–25. doi: 10.1037/a0017500, PMID: 21532933 PMC3083072

[58] Urquhart R Grunfeld E Jackson L Sargeant J Porter GA. Cross-disciplinary research in cancer: an opportunity to narrow the knowledge-practice gap. Curr Oncol (Toronto, ON). (2013) 20:e512–21. doi: 10.3747/co.20.1487, PMID: 24311951 PMC3851347

[59] Gibbs A Jacobson J Kerr WA. A global comprehensive review of economic interventions to prevent intimate partner violence and HIV risk behaviours. Glob Health Action. (2017) 10:1290427. doi: 10.1080/16549716.2017.1290427 28467193 PMC5645712

[60] White MC Peipins LA Watson M Trivers KF Holman DM Rodriguez JL. Cancer prevention for the next generation. J Adolesc Health. (2013) 52:S1–7. doi: 10.1016/j.jadohealth.2013.02.016, PMID: 23601606 PMC4402978

[61] World Health Organization. Global status report on violence prevention 2014. Geneva: World Health Organization (2014).

